# SuCComBase: a manually curated repository of plant sulfur-containing compounds

**DOI:** 10.1093/database/baz021

**Published:** 2019-02-22

**Authors:** Sarahani Harun, Muhammad-Redha Abdullah-Zawawi, Mohd Rusman Arief A-Rahman, Nor Azlan Nor Muhammad, Zeti-Azura Mohamed-Hussein

**Affiliations:** 1 Centre for Bioinformatics Research, Institute of Systems Biology (INBIOSIS), Universiti Kebangsaan Malaysia, UKM Bangi, Selangor, Malaysia; 2 Centre for Frontier Sciences, Faculty of Science and Technology, Universiti Kebangsaan Malaysia, UKM Bangi, Selangor, Malaysia

## Abstract

Plants produce a wide range of secondary metabolites that play important roles in plant defense and immunity, their interaction with the environment and symbiotic associations. Sulfur-containing compounds (SCCs) are a group of important secondary metabolites produced in members of the Brassicales order. SCCs constitute various groups of phytochemicals, but not much is known about them. Findings from previous studies on SCCs were scattered in published literatures, hence SuCComBase was developed to store all molecular information related to the biosynthesis of SCCs. Information that includes genes, proteins and compounds that are involved in the SCC biosynthetic pathway was manually identified from databases and published scientific literatures. Sets of co-expression data was analyzed to search for other possible (previously unknown) genes that might be involved in the biosynthesis of SCC. These genes were named as potential SCC-related encoding genes. A total of 147 known and 92 putative *Arabidopsis thaliana* SCC-related genes from literatures were used to identify other potential SCC-related encoding genes. We identified 778 potential SCC-related encoding genes, 4026 homologs to the SCC-related encoding genes and 116 SCCs as shown on SuCComBase homepage. Data entries are searchable from the Main page, Search, Browse and Datasets tabs. Users can easily download all data stored in SuCComBase. All publications related to SCCs are also indexed in SuCComBase, which is currently the first and only database dedicated to plant SCCs. SuCComBase aims to become a manually curated and *au fait* knowledge-based repository for plant SCCs.

## Introduction

Secondary metabolites are metabolites synthesized by living cells that are not directly involved in cellular metabolism of organisms such as growth, development and reproduction (1, 2). In plants, secondary metabolites act as chemical defense against plant pests and pathogens. Many experimental findings revealed various important functions of secondary metabolites indicating their role and importance in defense response against pathogens (1, 3–6). Apart from being toxic or repellant to herbivores or pests and microbes, they also regulate beneficial interactions such as attracting pollinators or seed dispersal and modulation of abiotic stress responses (7–9).

Different characteristic plant species–specific mix of these chemicals can be used as taxonomic identifier in plant classification (10, 11). Secondary metabolites are extremely diverse and usually belong to one of the three major classes, namely terpenes, phenolics and alkaloids (5, 12). However, there is one unusual plant constituent that was found to be highly involved in plant defense system known as sulfur-containing compounds (SCCs) (11). SCCs are very important in plant–pest interaction in various types of plant families comprising species-specific defense chemicals such as camalexin in Brassicaceae, glucosinolates (GSLs) in Brassicales, alline in Alliaceae, thiopene in Asteraceae and defensins in other plant families (13).

Currently, there are almost 200 SCCs found in Brassicaceae (11, 14). Camalexin for example, is a major phytoalexin SCC found in *Arabidopsis thaliana* that plays an important role in deterring pathogens such as *Botrytis cinera* (15) and *Alternaria brassicicola* (16). Research on SCCs has contributed to new knowledge on their important biological induction: for example, antioxidant activity, chemoprevention effects and apoptosis (17). However, among known SCCs, only GSLs and isothiocyanates have become popular research subjects due to their anticancer activities (18–22). Isothiocyanates in cruciferous vegetables were shown to induce glutathione S-transferase and NAD(P)H:quinone oxidoreductase 1 that act as cell protectants by detoxifying against potential carcinogens and oxidants (23), which explain their effect in reducing the risk of bladder cancer in individuals who consume loads of these vegetables (24, 25). Besides, they also have broad antibiotics properties such as antimicrobial, nematocidal, antifungal and antiprotozoal (26, 27). Furthermore, sulforaphane is one of the most studied isothiocyanates in numerous animal studies [e.g. samples taken from lung (28), colon (29), breast (30), skin (31), stomach (32), small intestine (33) prostate (34), pancreas (35) and oral cells (36)]. The findings reported the ability of sulforaphane to inhibit the carcinogenic cells at either in the early or late stages of malignant tissues. Meanwhile, GSLs are potential putative repellents and attractants of diamondback moth (DBM), *Plutella xylostella* L., which is a widespread destructive pest on Brassicales crops (37, 38). A number of studies revealed the contribution of different GSL profiles toward the behavior of DBM feeding that serve as novel findings in plant–herbivore interactions (39–41).

Information regarding SCCs genes, proteins and compounds that are involved in SCC biosynthetic pathway is abundant due to their potential contribution in pharmaceutical and agricultural industries. The availability of co-expression studies has significantly contributed to the search of new or potential SCC-related encoding genes. However, this information was distributed in various literature and biological databases, causing difficulties in finding all genes and molecular information of SCCs. This limitation has prompted the need to develop a digital repository that provides a platform for easy access of comprehensive information on SCCs, hence the development of a manually curated database called SuCComBase. The collective data in this database aims to provide valuable resources for genomic studies on potential SCC-related genes that might be involved in the SCC biosynthetic pathway. Herein, the development and current status of SuCComBase were described with the web interface systematically elaborated. SuCComBase is accessible at http://plant-scc.org (SuCComBase ver1.2, last updated on 4 January 2019).

## Materials and methods

### Data collection

Extensive bibliomic (all related publications published between early 2001 and 2017) and database searching from publication and biological databases were carried out to identify all genes responsible in encoding the SCCs related proteins in *A. thaliana*. Known SCC-related genes and compounds were identified using various keywords such as ‘sulfur containing compounds’, ‘sulfur containing secondary metabolites’, ‘sulphur containing compounds’, ‘sulphur containing secondary metabolites’, ‘glucosinolate’, ‘phytoalexin’, ‘camalexin’ and ‘Brassicaceae’, ‘*Arabidopsis thaliana*’, ‘*Brassica rapa’*, ‘cabbage’, ‘*Brassica oleracea*’, ‘broccoli’, ‘*Carica papaya*’ and ‘papaya’. These keywords were searched in publication databases such as PubMed, ScienceDirect and Scopus and biological databases, e.g. AraCyc v8.0 (42), KEGG v88.2 (43), KNApSAcK (last updated on 29 June 2018) (44) and PubChem v1.6.2 beta (45). Putative SCC-related genes were also identified using the keyword search in AraCyc and KEGG; however, no experimental evidence can be found to support their contribution in SCC biosynthesis.

**Figure 1:**
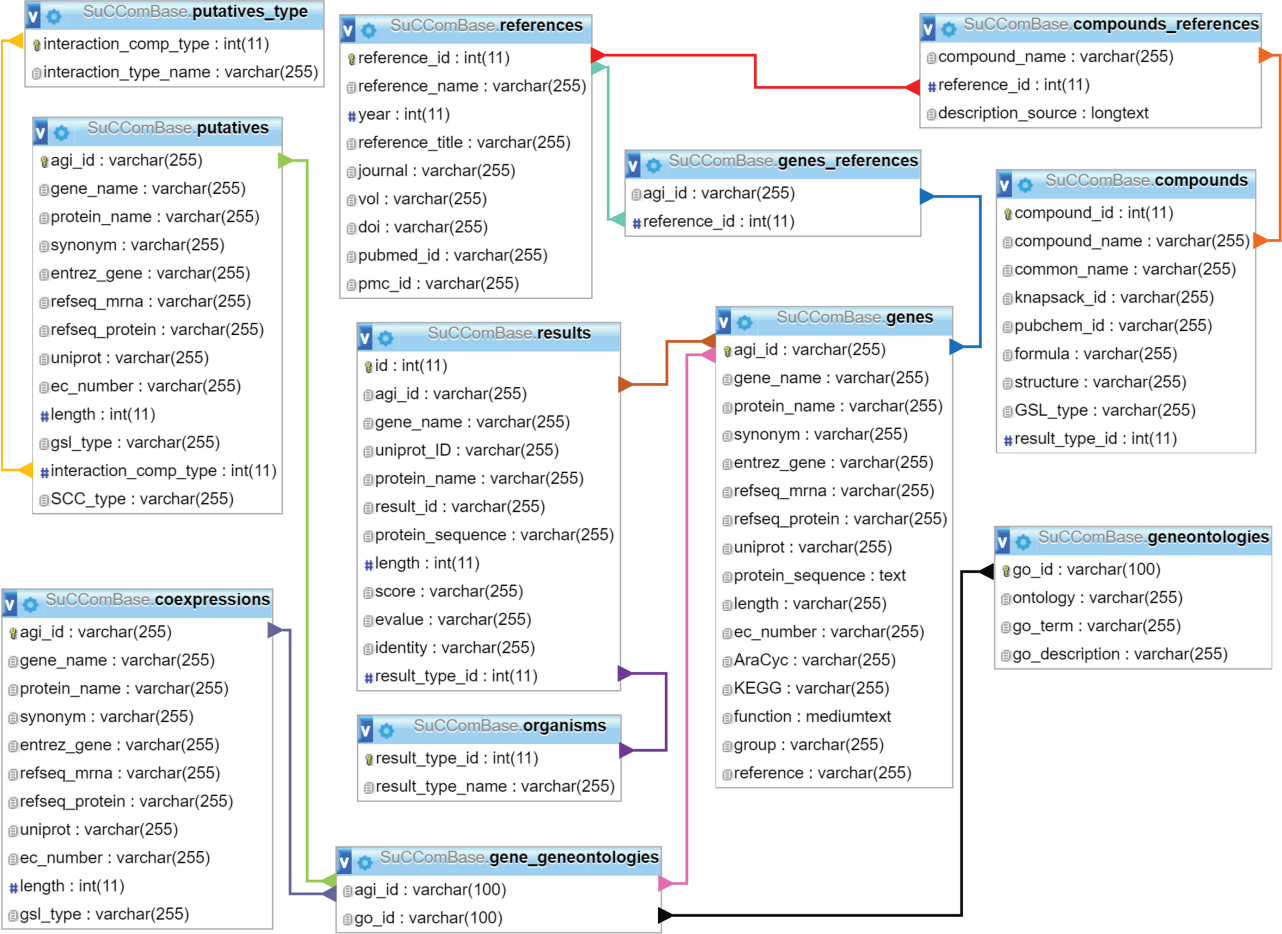
SuCComBase schema contains 12 tables with the connections from table to table.

### Functional annotation

Genes identified from database searching were manually curated using all relevant information obtained from various databases such as NCBI Gene (last updated 4 December 2018) (46), UniProt (last updated 5 December 2018) (47), AraCyc (42), KEGG (43), Ensembl Plants v41 (48), KNApSAcK (44) and PubChem (45). Three co-expression databases, i.e. ATTED v9 (49), AraNet v2 (50) and GeneMANIA (last updated on 14 March 2017) (51), were used to identify potential SCC-related encoding genes. The protein sequence of known SCC encoding genes of *A. thaliana* were retrieved from Phytozome v12.1.6, which is a Plant Comparative Genomics Portal containing 93 plant genomes (52). SCC homologs were identified via BLAST (53) against *B. rapa*, *B. oleracea* and *C. papaya* in Phytozome database using the known SCC protein sequences as queries. Gene Ontology Consortium (last updated on 26 October 2018) (54) was used to identify gene ontology (GO) in known SCC-related genes, potential SCC-related genes and putative SCC-related genes to provide a clearer understanding of SCC biosynthesis in *A. thaliana*.

### Database organization and architecture

SuCComBase consisted of 12 linked tables (Figure 1) with information on the SCC encoding genes and compounds, their functional information and references. MySQL Server 5.0.11 was used to host SuCComBase relational database. The SuCComBase web interfaces were developed using Laravel 5.3.31 (PHP web framework), HTML and JavaScript.

**Figure 2:**
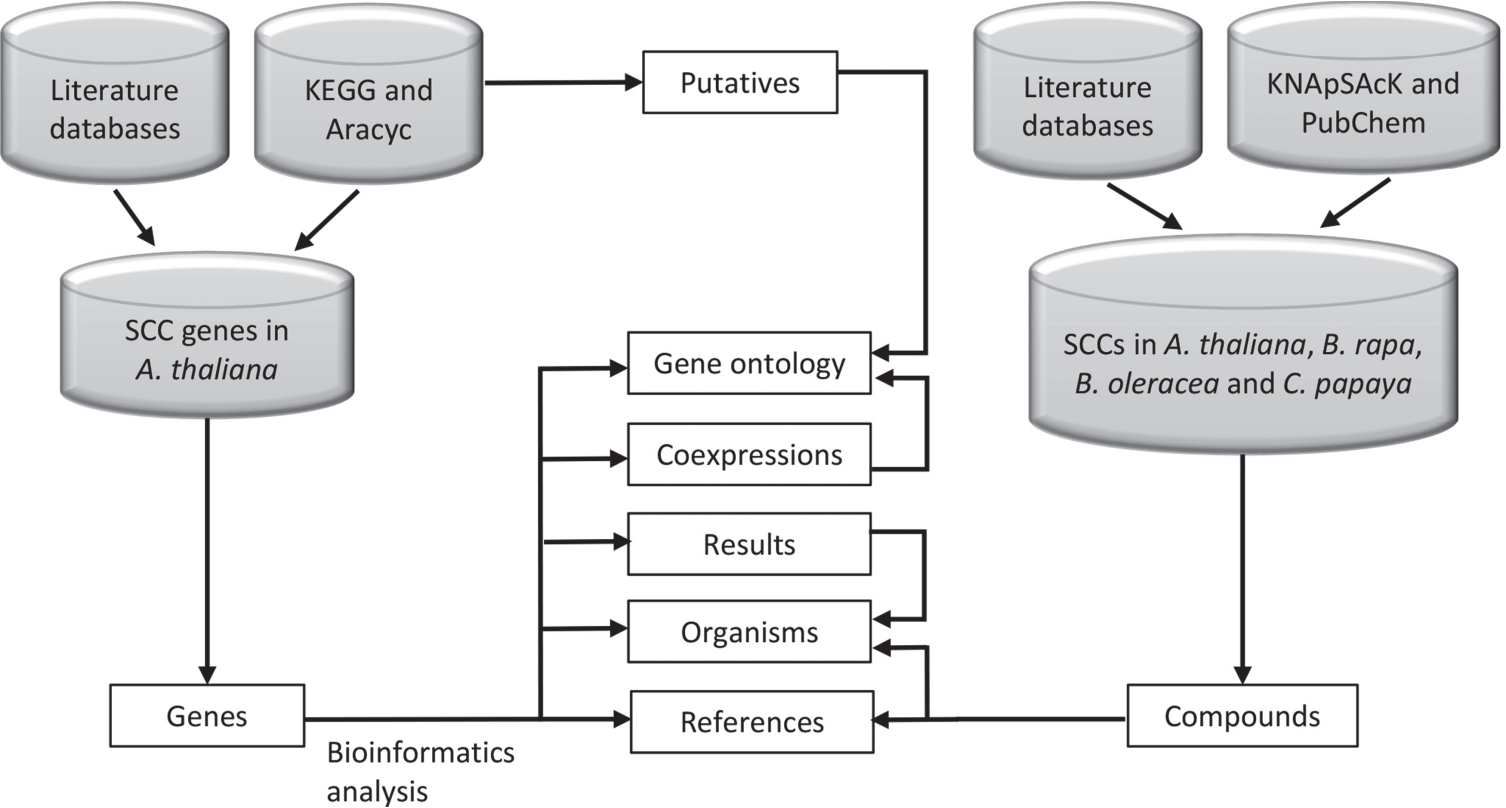
SuCComBase data types structure organization. These data types are tables that can be found in ‘Browse’ and ‘Datasets’ menu.

**Figure 3:**
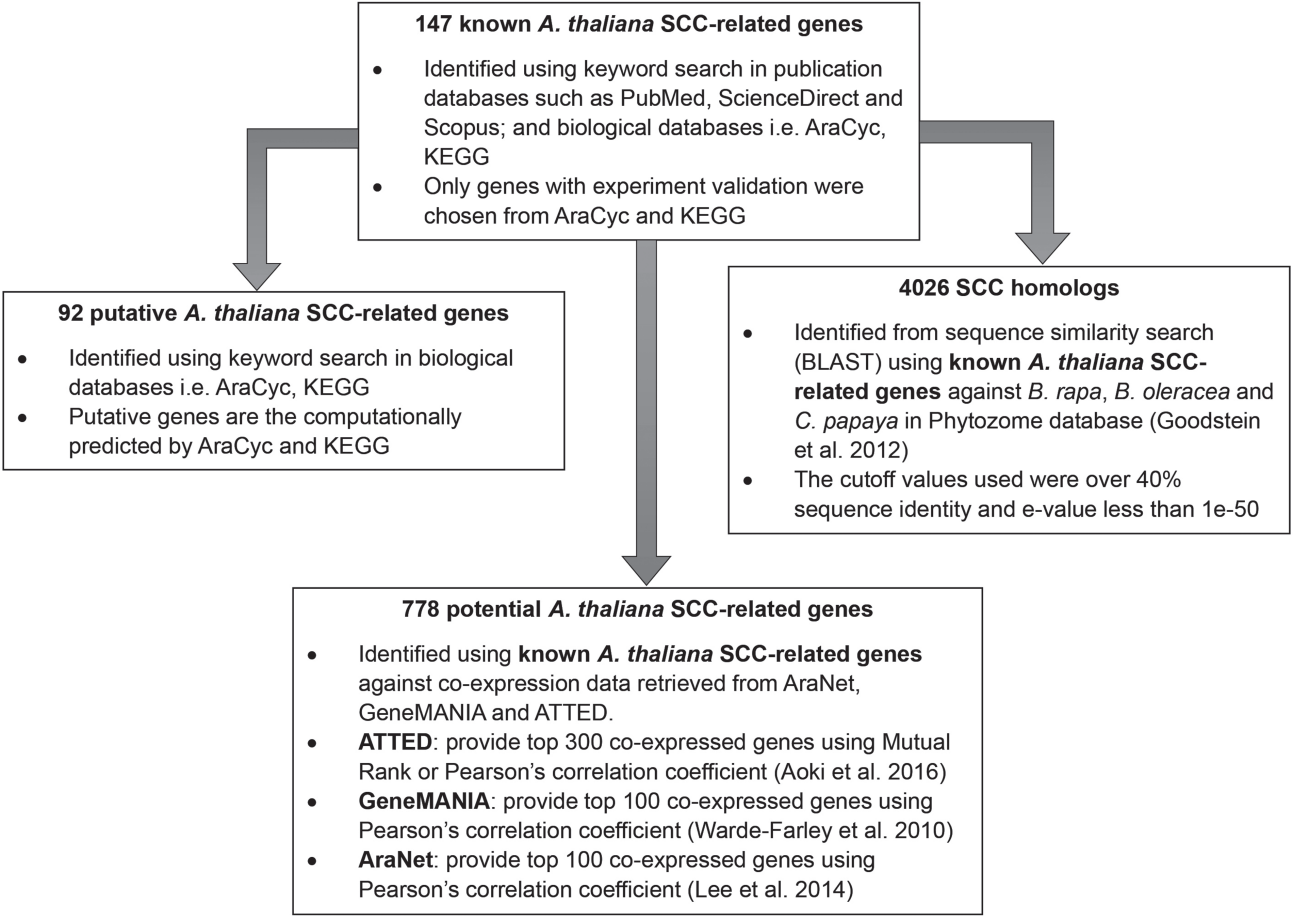
Identification of 92 putative *A. thaliana* SCC-related genes, 778 potential *A. thaliana* SCC-related genes and 4026 SCC homologs using 147 known *A. thaliana* SCC-related genes as queries.

**Figure 4:**
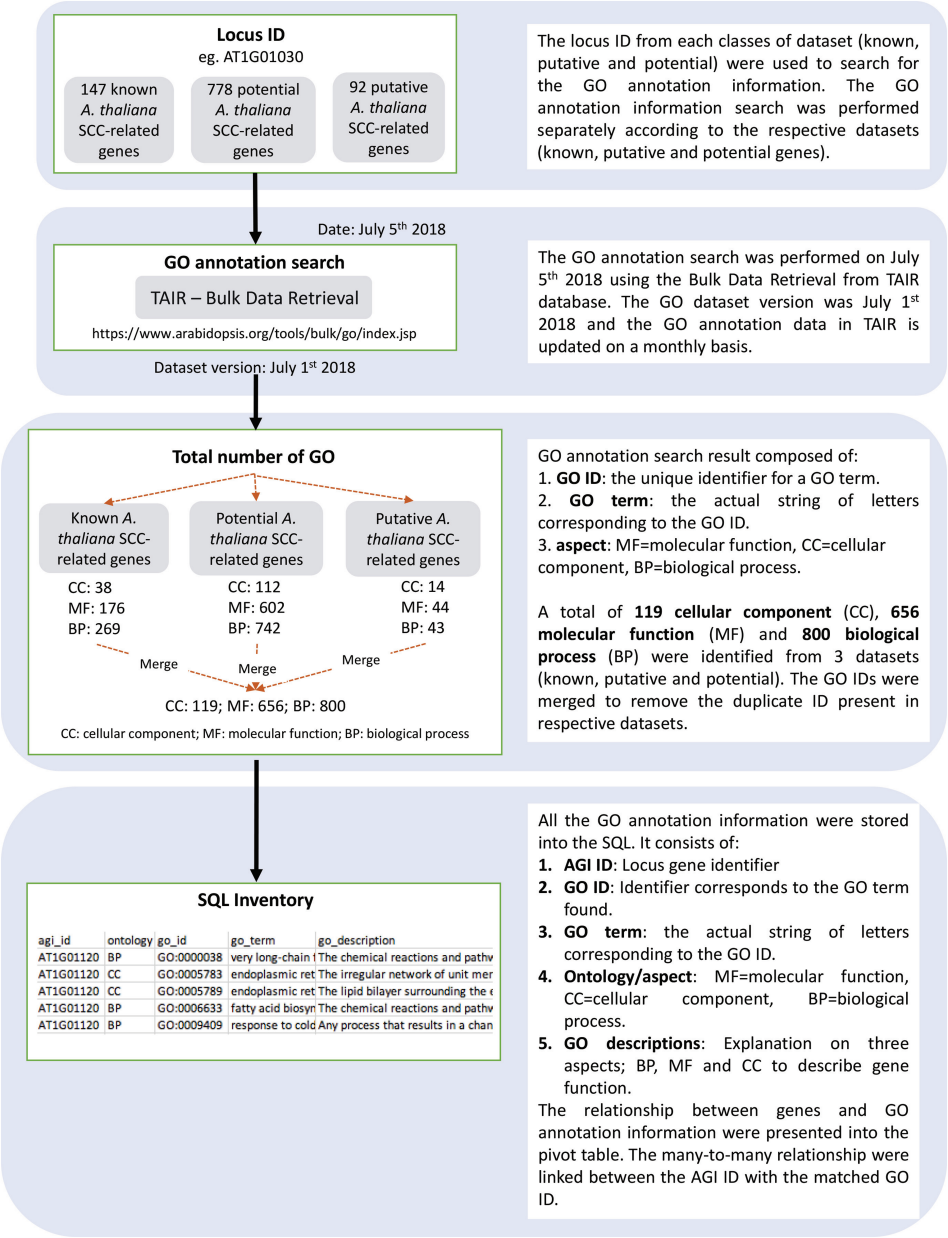
Detailed overview on the approaches used to identify and annotate GO terms to each SCC-related genes entry. Known, potential and putative *A. thaliana* SCC-related genes were used to search for the GO annotation information.

**Table 1:** Number of entries in SuCComBase

Data set	Entries
Known *A. thaliana* SCC-related genes	147
Putative *A. thaliana* SCC-related genes	92
KEGG putative *A. thaliana* SCC-related genes	3
AraCyc putative *A. thaliana* SCC-related genes	89
Potential *A. thaliana* SCC-related genes	778
SCC homologs	4026
*B. rapa* SCC homologs	1970
*B. oleracea* SCC homologs	1319
*C. papaya* SCC homologs	737
Compounds	116
*A. thaliana* SCCs	47
*B. rapa* SCCs	28
*B. oleracea* SCCs	40
*C. papaya* SCCs	1
Publications	206

**Figure 5:**
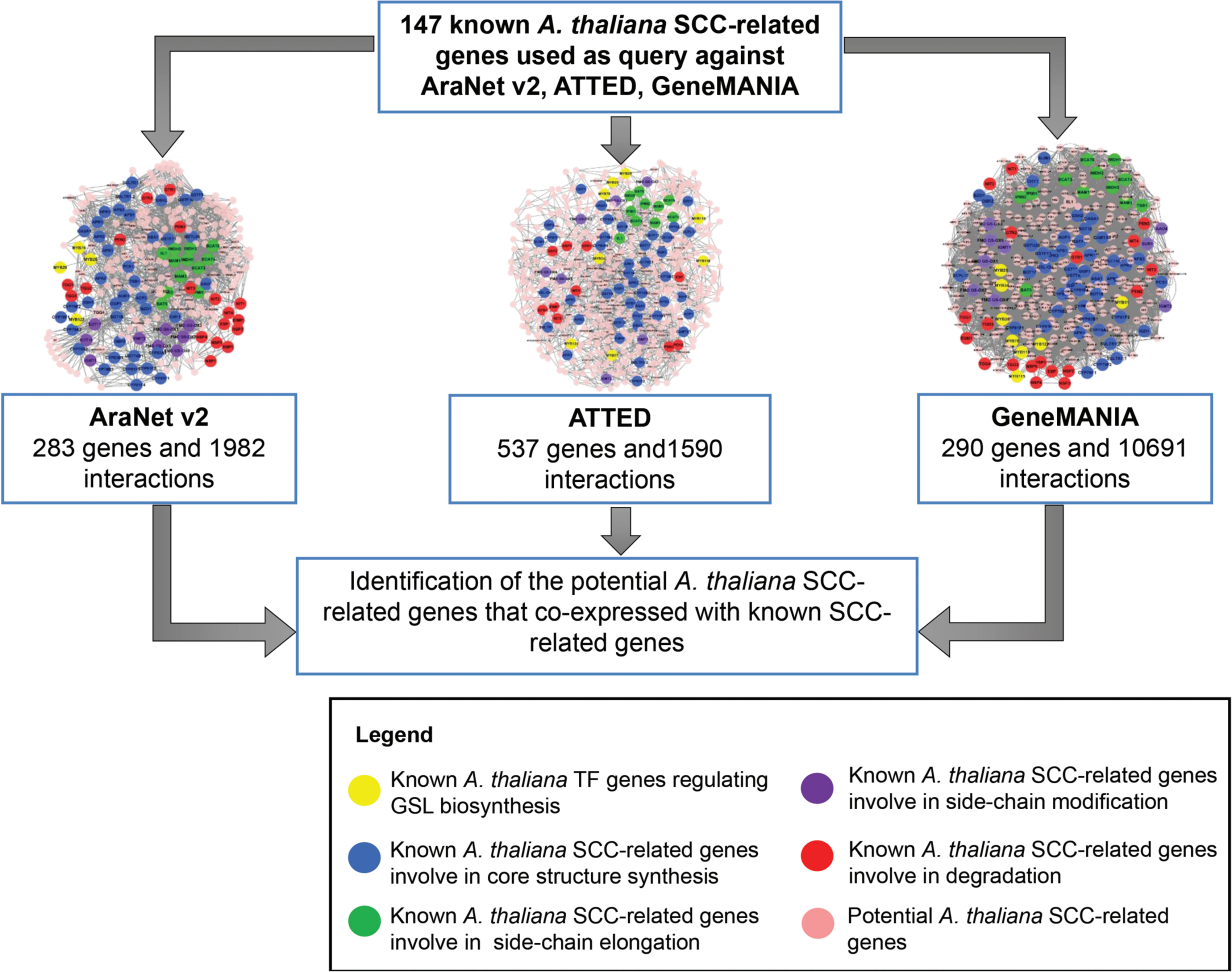
The integration of three co-expression gene networks reveals potential SCC-related genes. Different colors refer to the function of known SCC-related genes in GSL biosynthesis: yellow (transcription factor), blue (core structure synthesis), green (side-chain elongation), purple (side-chain modification) and red (GSL degradation). Known SCC-related genes were used as query to identify the co-expressed genes to be classified as potential SCC-related genes.

## Results and Discussion

### Database summary

Figure 2 shows the organization of data types whereby SCC-related genes in *A. thaliana* are assigned as the main data in SuCComBase. A total of 147 known SCC-related genes were manually curated and supported with added information obtained from KEGG and AraCyc. We have identified 92 computationally predicted genes that might be involved in the production of SCCs and classified them as putative SCC-related genes. The known SCC-related genes were used as queries in identifying potential SCC-related genes from the co-expression data, and 778 potential SCC-related genes were successfully identified from three co-expression databases: e.g. ATTED (49), AraNet (50) and GeneMANIA (51). BLAST analysis against Phytozome database has identified 4026 SCC homologs from three Brassicales plant genomes, i.e. 1970 SCC homologs in *B. rapa*, 1319 SCC homologs in *B. oleracea* and 737 SCC homologs in *C. papaya.*Figure 3 summarizes the steps involved in identifying putative *A. thaliana* SCC-related genes, potential *A. thaliana* SCC-related genes and the SCC homologs. Furthermore, we have included GO terms of the SCC-related genes where a total of 800 biological process, 656 molecular functions and 119 cellular components were included in this database. Figure 4 shows the steps performed in identifying the GO terms and the results obtained from the analysis. We have also identified 116 SCCs in *A. thaliana, B. rapa*, *B. oleracea* and *C. papaya* and included them in SuCComBase. Table 1 shows the summary of each data set in SuCComBase.

### Database interface and access

The interface of SuCComBase contains a homepage and seven main menus, i.e. About, Browse, Search, Datasets, Download, Help and Contact. These menus are used to facilitate the users in navigating the pages effortlessly.

(i) SuCComBase Homepage displays data statistics in each table and provides a brief overview of this database.
(ii) The background of SuCComBase can be viewed in the ‘About’ page.
(iii) The ‘Browse’ page allows users to assess all six data sets in SuCComBase. Each data set varied based on their biological information as described below:
  (a) Known *A. thaliana* SCC-related gene data set: contains SCC-related encoding genes in *A. thaliana* including protein-encoding genes that involve in the GSL and camalexin biosynthetic pathways based on experiments reported in various publications and pathway databases (KEGG and AraCyc).
  (b) Putative *A. thaliana* SCC-related gene data set: contains computationally predicted GSL and camalexin genes from KEGG and AraCyc databases.
  (c) Potential *A. thaliana* SCC-related gene data set: contains 778 *A. thaliana* genes that might be involved in the SCC biosynthetic pathway based on the bioinformatic analysis using co-expression data retrieved from AraNet, GeneMANIA and ATTED. Figure 5 shows an example of the network constructed between the potential SCC-related encoding genes.
  (d) SCC homolog data set: contains SCC homologs in *C. papaya*, *B. rapa* and *B. oleracea.*
  (e) Compound data set: contains SCCs produced in *A. thaliana*, *C. papaya*, *B. rapa* and *B. oleracea*. All information was obtained from extensive literature search and from KNApSAcK as well as PubChem databases.
  (f) Publication data set: contains 206 published articles that were used in identifying SCCs and all SCC-related encoding genes.
(iv) The ‘Search’ page is used to search for genes, compounds or any biological term that match to a particular keyword. For example, if ‘sulfur’ keyword is searched, all entries (Genes, Putative genes, Potential genes, SCC homologs, Compounds, Publications) in SuCComBase that contain ‘sulfur’ will appear.
(v) The ‘Datasets’ dropdown menu provides links to all data sets in SuCComBase. These Dataset tabs are located at the header of SuCComBase to help users navigate the database.
(vi) The ‘Download’ page provides access to current and archived data sources in SuCComBase.
(vii) The ‘Help’ menu contains manual of SuCComBase, database schema, data sources and all references used to retrieve the information in SuCComBase. All scientific terms, definition and FAQs that are related to SuCComBase were also provided in the ‘Help’ page.
(viii) The ‘Contact’ menu provides information on the SuCComBase developer contacts and email address.

## Conclusion and future work

SuCComBase is publicly available online at http://plant-scc.org and will be periodically updated. Currently, SuCComBase is the first and only database that provides the information on SCCs that are related to plant defense system in Brassicales. All information provided in this database is important to plant scientists, synthetic biologists, systems biologists, chemists or anyone who are interested working on the secondary metabolites or potential compounds, as well as those who study plant–host interactions specifically in Brassicales crops, hence continuous comprehensive cataloguing and curation is a priority.

## Notes

**Database URL**: http://plant-scc.org
